# Hip effusion/synovitis influences results after multiple drilling core decompression for bone marrow edema syndrome of hip

**DOI:** 10.1186/s12893-023-02066-8

**Published:** 2023-06-03

**Authors:** Hua-zhang Xiong, Yan-li Peng, Yu-hong Deng, Ying Jin, Ming-hong Tu, Shu-hong Wu

**Affiliations:** 1 Department of Orthopedic Surgery, Affiliated Hospital of Zunyi Medical University, 149# Dalian Road, Zunyi, Guizhou Province 563003 People’s Republic of China; 2 Department of Orthopedic Surgery, Renhuai Hospital of Traditional Chinese Medicine, Renhuai, Guizhou, Guizhou Province 564500 People’s Republic of China

**Keywords:** Hip effusion, Synovitis, Arthroscopy, Multiple drilling core decompression, Treatment, Bone marrow edema syndrome

## Abstract

**Background:**

At present, it is not known whether hip effusion/synovitis affects the therapeutic effect of multiple drilling core decompression (MDCD) in patients with bone marrow edema syndrome of hip (BMESH). The aims were to assess hip effusion/synovitis and its relationship with results of MDCD in patients with BMESH.

**Methods:**

The data of undergoing arthroscopic-assisted MDCD for treatment of BMESH with hip effusion/synovitis by one surgeon were retrospectively reviewed from the associated medical records at the Affiliated Hospital of Zunyi Medical University (2016–2019). Seven patients (9 hips) participated in this study. Patients were followed up at 1, 2, 3, 6, 12 and 24 months. Data included demographics and clinical outcomes. The pre- and postoperative pain and functional outcomes were measured with the visual analogue scale (VAS), Harris Hip Score (HHS), Hip Outcome Score Activities of Daily Living subscale (HOS-ADL), International Hip Outcome Tool-12 (iHOT-12) and range of motion (ROM).

**Results:**

Seven patients (9 hips) were followed up. Disappearance of hip pain immediately obtained at rest after surgery. All of 7 patients returned to their former activity level at postoperative 3 months, bone marrow edema had disappeared on Magnetic Resonance Imaging (MRI). The VAS, HHS, HOS-ADL, iHOT-12, and ROM at postoperative 1 month had a significant difference (*P < 0.05*) compared with preoperative. It was also statistically significant (*P < 0.05*) when compared with other time points. At the final follow-up, all patients had no limited ROM, which was symmetrical with the contralateral of hip joint. Hip effusion/synovitis were observed in 9 hips. Labral tears, cartilage fissure, and loose bodies were observed in 1 hip, respectively. Kirschner wire tracks bleeding occurred in 1 hip. No other complications occurred.

**Conclusions:**

Hip effusion/synovitis could affect the clinical outcomes after MDCD in patients with BMESH. Arthroscopic procedure of hip effusion/synovitis can shorten postoperative pain relief time, disappearance time of bone marrow edema on MRI. It can simultaneously diagnose and treat other concomitant intraarticular pathologies, and be a safe operation with fewer complications.

## Introduction

**Bone marrow edema syndrome of hip (BMESH) is a self-limited disease characterized by pain and limited range of motion (ROM) that mainly involves in the femoral head and neck** [1]. Magnetic resonance imaging (MRI) shows hypointense area on T1-weighted sequences and a hyperintense area on T2-weighted sequences [2]. Bone marrow edema syndrome (BMES) was first defined by Curtiss et al. in 1959 [3]. The etiology and pathogenesis of BMES still remain unclear. The pathology usually resolves spontaneously after pain persists for approximately 3 to 24 months [1, 2, 4]. BMESH is scarce but likely complication could be the progression to osteonecrosis of femoral head (ONFH) [5].

The current discovery of the pathogenesis considers primarily BMESH as a pathology of bone. The preferred treatment of BMESH remains controversial. Treatment of BMESH is mainly dependent on the severity of pain and the requirements of the patient because BMESH is a self-limited disease. Treatment usually consists of observation, non- and surgical treatment [6, 7]. Because BMESH can spontaneously cure, asymptomatic entity can be treated through observation. Non-surgical therapies include extracorporeal shock wave [1], bisphosphonates [8, 9], vasoactive drugs (Iloprost) [5, 6], and hyperbaric oxygen [10]. These therapies have been revealed to alleviate pain, but pain relief time and functional recovery time are uncertain. The pain relief time varies widely, which ranged from several days to 6 months with the majority between 1 month and 6 months. Very few pain relief times were from several days to three weeks, such as using Iloprost therapy. But the patients may suffer adverse effects, it has some strict indications and contraindications with narrow range of use [5, 6].

**Surgical treatment consists of two main methods: traditional core decompression (CD)** [11] **and multiple drilling core decompression (MDCD)** [12]. CD through drilling a hole in the affected site allows to decrease intramedullary pressure and increase blood supply. **CD has been applied in the clinic over many years and may be considered for improvement of symptoms and reduction of MRI changes** [6]. The MDCD was performed with femoral head decompression through the lateral approach to the hip. A Kirschner pin was placed 2 to 5 cm inferior to the greater trochanter, then drilled at least 10 times into the involved area of the femoral head and neck [4]. **MDCD has achieved good results with lower complications, such as subtrochanteric fracture** [4].

Those studies have reported that the surgical techniques could shorten disease course. **However, few attentions have been paid to intraarticular conditions, such as hip effusion/synovitis** [13] **in patients with BMESH**. Hip effusion/synovitis are thought to be secondary to the bone marrow edema, where the intraosseous hypertension causes disturbance of blood circulation and reactive hip/effusion synovitis [14]. **Hip effusion/synovitis may be a source of hip pain in patients with BMESH** [15]. It is unknown whether hip effusion/synovitis has relationships with postoperative results of surgery. Furthermore, detailed intraarticular lesions in patients with BMESH are also unclear. With this limited body of studies, another method, termed arthroscopic-assisted MDCD, was developed. Arthroscopic-assisted MDCD has the advantages of having a decompression, and having the ability to simultaneously detect and manage the intraarticular pathologies such as hip effusion/synovitis, labral tears and loose bodies [16, 17]. To our knowledge, outcomes of arthroscopic-assisted MDCD for treating BMESH with hip effusion/synovitis has been not reported. The aim of this study is to retrospectively evaluated and analyse the clinical and follow-up data of arthroscopic-assisted MDCD for treating BMESH with hip effusion/synovitis and provide a reference for clinical management.

## Materials and methods

This was a retrospective study to assess the outcomes of the arthroscopic-assisted MDCD for treatment of BMESH with hip effusion/synovitis. The study was approved by the Ethics Committee of the Affiliated Hospital of Zunyi Medical University (KLL-2022-696). All methods were performed in accordance with the relevant guidelines and regulations. All operations in this study were performed through arthroscopic-assisted MDCD by one senior surgeon between January 2016 and December 2019. All of patients diagnosed with BMESH associated with hip effusion/synovitis were subsequently identified. All patients were evaluated by the senior author and accepted a standard preoperative examination for patients undergoing an arthroscopic-assisted MDCD, including hip MRI and supine anteroposterior views of hip joint to assess the extent of pathologies. Preoperative MRI allowed for the diagnosis of hip effusion/synovitis and other concomitant intra-articular pathologies, including loose bodies, labral tear, femoroacetabular impingement, and cartilage flaps. The concomitant intraarticular pathologies influence the decision to undergo hip arthroscopy because the primary indication for operation was arthroscopic management of hip effusion/synovitis.

**Fig. 1:**
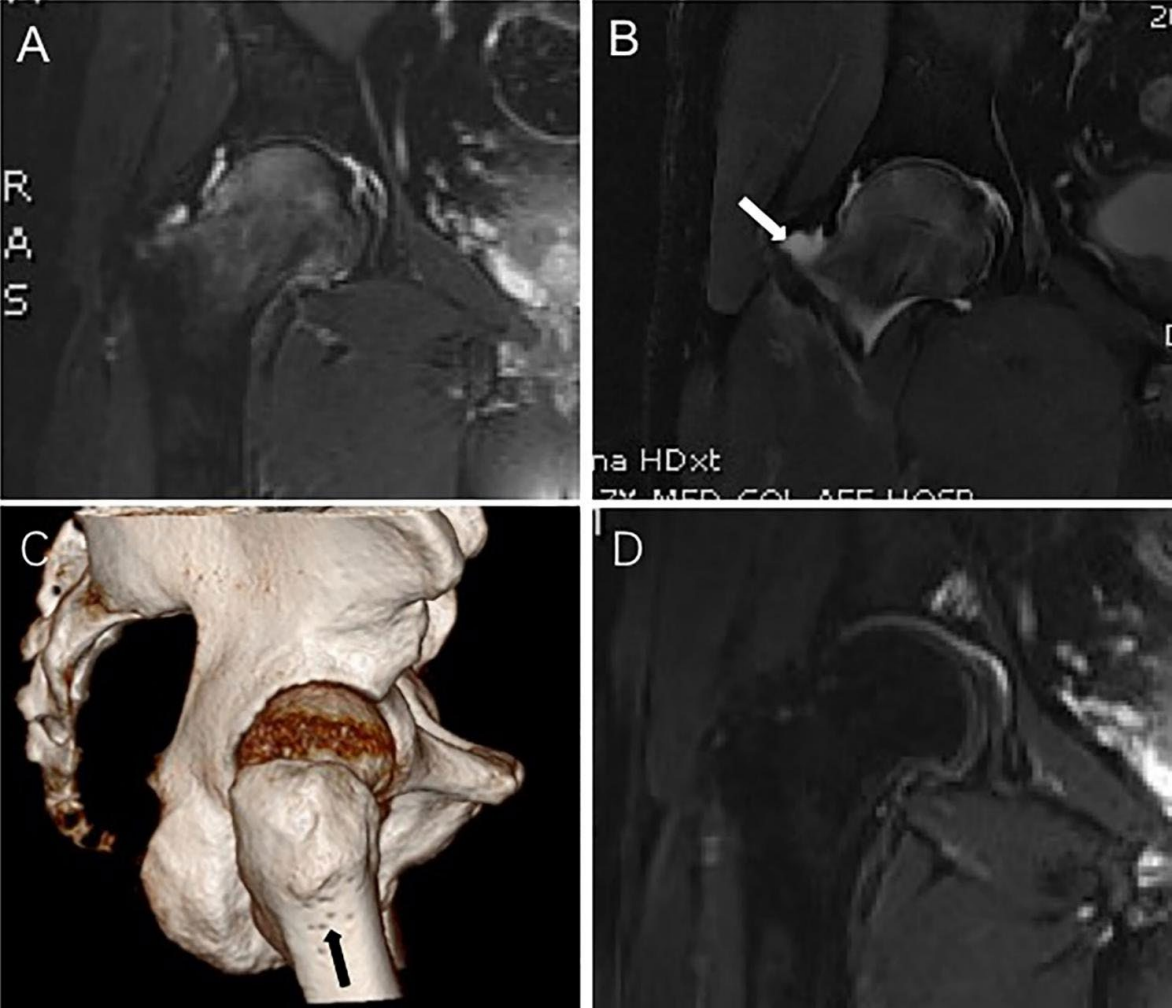
**A** Preoperative MRI T2-weighted coronal image of right hip demonstrated a bone marrow edema of femoral head and neck. **B** MRI T2-weighted coronal image of right hip showed an effusion pattern of the hip cavity (white arrow). **C** Postoperative 3D imaging showed the tracks of multiple drilling decompression at lateral side of proximal femur (black arrow). **D** Postoperative MRI T2-weighted coronal image of right hip demonstrated disappearance of bone marrow edema of femoral head and neck

We chose the surgery for the following indications. Conservative treatment of nonsteroidal anti-inflammatory drugs (NSAIDS) met failure. Hip joint was painful and range of motion (ROM) was severely affected. The patient was unwilling to be treated conservatively and required surgical treatment to shorten the course of the disease. Pain was exacerbated to affect sleep during night. All patients with BMESH combined with hip effusion/synovitis were clearly diagnosed by preoperative MRI (Fig. 1A, B). Contraindications of operation included following conditions. Treatment of NSAIDS was effective. BMESH was asymptomatic. BMESH was secondary, such as bone bruise, micro- or stress fracture, tumor, osteomyelitis, and osteoarthritis. BMESH combined with ONFH. Other causes leaded to joint effusion, such as infection, gout, pseudogout.

A total of 7 patients (9 hips) treated were analysed, including 5 males and 2 females. The average age was 48.9 ± 7.1 years (range, 42–59). There were 4 hips of an involved right side and 5 hips of an involved left side. To assess the prognosis of the BMESH, MRI was used at postoperative 3 months. The Harris Hip Score (HHS), Hip Outcome Score Activities of Daily Living subscale (HOS-ADL), International Hip Outcome Tool-12 (iHOT-12), and ROM were used for evaluating hip function. The hip pain evaluation proposed by the visual analogue scale (VAS) score was also used. The HHS was applied to assess postoperative recovery hip function. A total score of 91–100 is defined as an excellent score, 81–90 as good, 71–80 as fair, and < 70 as poor. The VAS was applied to assess pain on a scale from 0 to 10 (0 = no pain, and 10 = worst pain ever).

### Operative technique

**Fig. 2:**
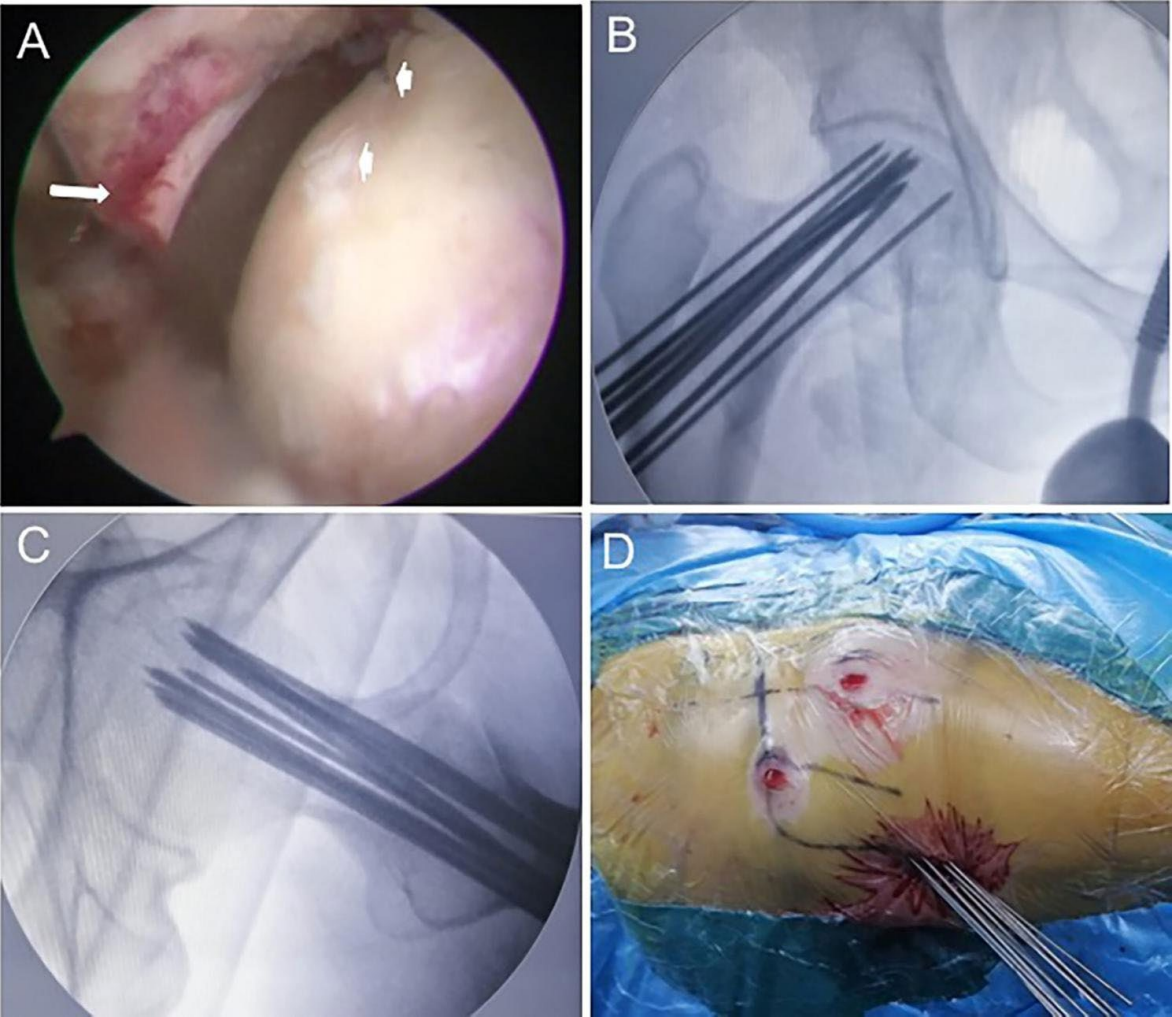
**A** Intraoperative imaging showed the congested synovium (white arrow) and cartilage fissure of femoral head (white arrows). **B, C** Intraoperative radiographic imaging showed pattern of multiple drilling decompression at proximal femur. **D** Intraoperative imaging showed percutaneous multiple drilling decompression of proximal femur

All of patients underwent arthroscopic-assisted MDCD under general anesthesia through the anterolateral (AL) and mid-anterior (MA) portals in the supine position on the traction table. The arthroscopic surgery to the hip was applied to establish portals according to outside-in fashion and “touch method” without applying distraction [18]. The AL portal was made after making a vertical stab incision, the subcutaneous and muscular layers were split by a straight clamp. The straight clamp was directed medially, pointing in the direction of the femoral neck. When the tip of the straight clamp touched femoral neck it was replaced with a blunt trocar and the femoral neck could be felt. The tip of the trocar was situated extra-articular at the femoral neck of the hip joint. The MA portal was made after making the skin incision, a straight clamp was introduced and directed toward the arthroscope trocar. When the straight clamp touched the trocar of the arthroscope, the trocar was used as a guide to travel medially in the direction of the femoral neck. The straight clamp must touch the arthroscope trocar until the straight clamp touches bone. An ablator or shaver was introduced. After the fatty tissue of the pre-capsule was removed with shaver the white structure of the joint capsule was showed. Then an extra-articular longitudinal capsulotomy was performed from outside to inside. A diagnostic arthroscopy of the affected hip was performed in order to assess the presence or absence of the concomitant intraarticular pathologies under distraction. It was addressed if the synovitis was observed (Fig. 2A). If other intraarticular pathologies were present, the related procedure was performed. After the diagnosis and treatment of the concomitant intraarticular pathologies, the arthroscope was removed and the traction was released.

Under C-arm fluoroscopy, multiple 2.0 -mm-diameter Kirschner wires were drilled from the proximal lateral femur cortex through the femoral neck and femoral head to perform CDs on demand (Fig. 1C and Fig. 2B, C, D). Intraoperative fluoroscopy was used to confirm sufficient decompressions at the edematous area.

All individuals received postoperative prophylactic anti-thrombotic management. We allowed the patient to perform partial weight bearing using crutches for 6 weeks postoperatively. The operative site of the hip was protected against any unexpected twisting for 3 months. The patient was followed up at 1, 2, 3, 6, 12 and 24 months. The preoperative and postoperative VAS, HHS, HOS-ADL, iHOT-12 and ROM were separately evaluated and analysed to compare the differences between the preoperative and postoperative. The postoperative pain relief time, disappearance time of bone marrow edema, and complications were recorded.

### Statistical analyses

Statistical tests were performed using SPSS software SPSS® version 22 (SPSS Inc., Chicago, Illinois). All values were expressed as the mean values with standard deviations (SDs). The normally distributed data were tested using parametric independent t-tests, and Wilcoxon tests were performed to test the non-normally distributed data regarding the pre- and postoperative outcomes (VAS, HHS, HOS-ADL, iHOT-12 score and ROM). A difference in value was considered to be statistically significant when the corresponding p-value was *< 0.05*.

## Results

A total of 9 THAs (7 patients), who underwent the arthroscopic-assisted MDCD for BMESH with hip effusion, were retrospectively reviewed and included. The demographic data in terms of age, sex, body mass index (BMI), and affected side showed in Table 1.

**Table 1:** Demographic and characteristics of patients

Pa	Age	Ge	BMI	DPP	OT	PHS
1	59	Ma	25.8	8	45	1
2	47	Ma	22.0	4	50	2
3	42	Fe	25.1	5	42	2
	43	Fe	25.3	7	53	3
4	52	Ma	21.3	3	47	1
5	39	Ma	27.3	5	56	2
6	55	Ma	21.8	6	48	2
	57	Ma	22.9	13	54	2
7	46	Fe	25.0	6	51	1
	48.9 ± 7.1		24.1 ± 2.1	6.3 ± 1.0	49.6 ± 4.5	1.78 ± 0.2

Note. BMI: Body Mass Index; DPP: Duration of Preoperative Pain (weeks). Fe: Female; Ge: Gender; Ma: male; OT: Operating Time (min); Pa: Patient; PHS: Postoperative Hospital Stays (days)

**Fig. 3:**
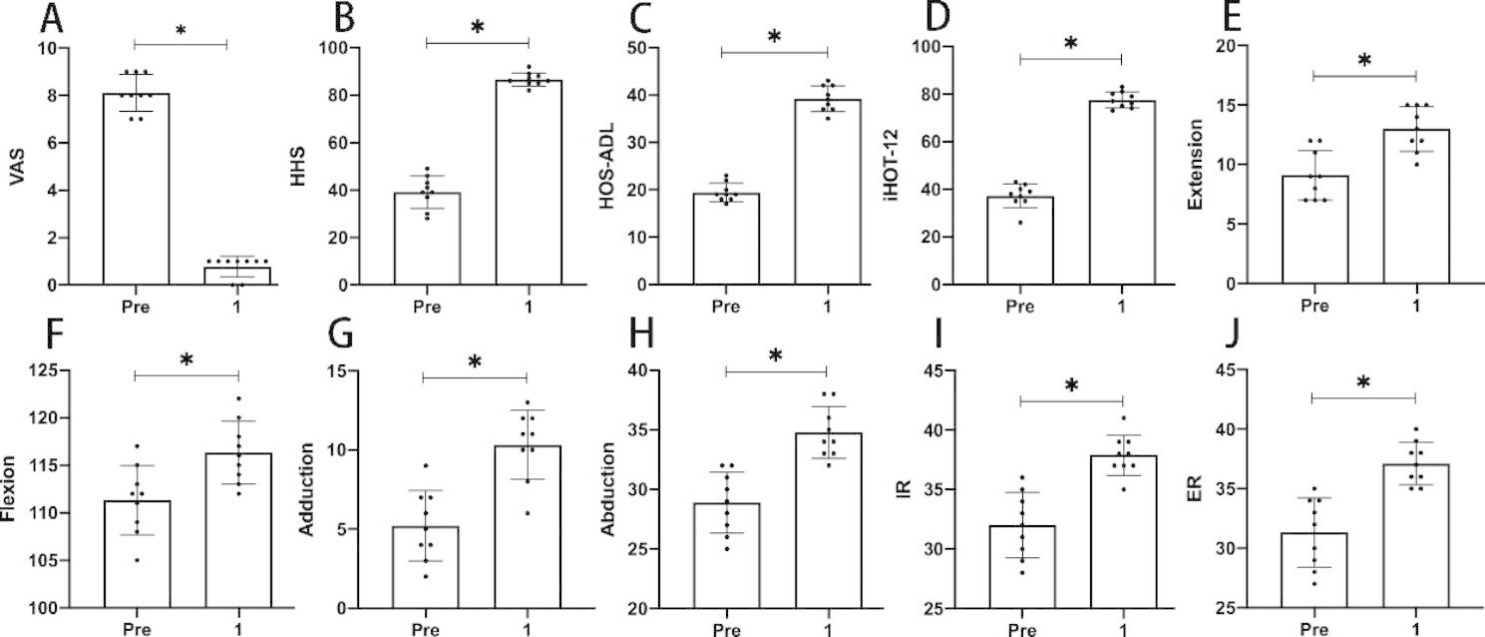
Results of VAS, HHS, HOS-AL, iHOT-12, and ROM at preoperative and one month after surgery. ER, External Rotation; HHSs, Harris Hip Scores; HOS ADL, Hip Outcome Score Activities of Daily Living Subscale; iHOT-12, International Hip Outcome Tool-12; IR, Internal Rotation; M, Months; Pre, Preoperative. Post, Postoperative; VAS, Visual Analogue Score

Disappearance of hip pain immediately obtained at rest after operation. At postoperative 3 months, all of 7 patients returned to their former activity level, MRI showed that bone marrow edema had disappeared (Fig. 1D). All patients had no limited ROM at postoperative 24 months, which was symmetrical with the contralateral of hip joint. The VAS, HHS, HOS-ADL, iHOT-12, and ROM at postoperative 1 month had a significant difference (*P < 0.05*) compared with preoperative in Table 2 and Fig. 3. It was also statistically significant (*P < 0.05*) when compared with other time points. The excellent rate of HHS was 100% at postoperative 24 months. Hip effusion/synovitis were observed in 9 hips in Table 3. Labral tear, cartilage fissure, and loose bodies were observed in 1 hip, respectively. Kirschner wire tracks bleeding occurred in 1 hip, and the bleeding stopped through cryotherapy and compression therapy after 2 dressing changes. There were no other postoperative complications, including deep vein thrombosis (DVT), sub-trochanteric fracture or arthroscopy-related complications in any patient.

**Table 2:** Outcomes of the hip at the preoperative and postoperative follow-up

	Pre	Post 1 M	Post 2 M	Post 3 M	Post 6 M	Post 12 M	Post 24 M
VAS	8.1 ± 0.8	0.8 ± 0.4*	0.1 ± 0.3	0.0 ± 0.0	0.0 ± 0.0	0.0 ± 0.0	0.0 ± 0.0
HHS	40.6 ± 7.0	86.7 ± 2.8*	91.1 ± 3.8	93.1 ± 3.6	94.0 ± 3.0	95.4 ± 1.6	96.3 ± 1.0
HOS-ADL	28.6 ± 2.8	57.7 ± 4.0*	74.5 ± 2.6	82.2 ± 2.6	89.9 ± 4.1	95.9 ± 2.1	98.4 ± 1.1
iHOT-12	31.0 ± 4.2	64.6 ± 2.8*	68.0 ± 2.2	72.3 ± 1.7	75.6 ± 2.2	81.3 ± 3.1	83.9 ± 2.5
Ex (°)	2.56 ± 2.6	9.1 ± 2.1*	13.0 ± 1.9	14.1 ± 0.9	14.4 ± 0.5	14.6 ± 0.5	14.8 ± 0.4
Fl (°)	111.3 ± 3.6	116.3 ± 3.8*	119.7 ± 3.8	122.1 ± 2.7	124.2 ± 2.0	124.6 ± 1.6	125.1 ± 1.2
Ad (°)	5.2 ± 2.2	10.3 ± 2.2*	13.9 ± 1.9	16.4 ± 1.0	17.7 ± 1.0	18.4 ± 1.1	19.2 ± 1.0
Ab (°)	28.9 ± 2.6	34.8 ± 2.2*	37.1 ± 1.6	38.7 ± 2.0	39.6 ± 1.9	40.3 ± 1.5	42.4 ± 1.1
IR (°)	32.0 ± 2.7	37.9 ± 1.7*	39.9 ± 1.5	40.9 ± 1.3	41.7 ± 1.0	42.4 ± 0.9	44.3 ± 1.0
ER (°)	31.3 ± 2.9	37.1 ± 1.8*	39.0 ± 1.3	40.3 ± 1.1	41.3 ± 1.2	42.6 ± 0.5	44.2 ± 1.2

Values are means (± SD). *: *p < 0.05* (t-test)

Note. Ab: Abduction; Ad: Adduction; ER, External Rotation; Ex: Extension; Fl: Flexion; HHS, Harris Hip Score; HOS ADL, Hip Outcome Score Activities of Daily Living Subscale; iHOT-12, International Hip Outcome Tool-12; IR, Internal Rotation; M, Months; Pre, Preoperative. Post, Postoperative; VAS, Visual Analogue Score.

**Table 3:** Concomitant pathologies at hip central compartment

Concomitant Pathologies	N (hip)	Treatment
hip effusion/Synovitis	9	synovectomy
Labral tear	1	labral debridement
Loose bodies	1	loose bodies removal
Cartilage fissure	1	debridement

## Discussion

It is still controversial with respect to the pathogenesis of BMES, and it shows the lack of an optimal treatment in this disease. **The rationale for using arthroscopic-assisted MDCD to treat BMESH originates from the clinical results of the management regarding the mechanism of action of arthroscopic-assisted MDCD on ONFH** [17]. **The clinical outcomes that reported in ONFH have similar characteristics to BMESH** [19]. MDCD have already been applied in the management of ONFH, which have been demonstrated to improve the healing of the necrotic site and obtain reliable results [17]. **There was histological evidence for an ischemic etiology of BMESH, which could be a reversible form of ONFH** [20]. **The suspected common pathophysiological pathway between BMESH and ONFH makes CD a therapeutic option** [21]. Therefore, in a similar manner it is possible that MDCD may significantly alleviate pain and resolve the BMESH. The purpose of MDCD includes to reduce intramedullary pressure, increase blood supply and relieve pain, shorten natural course of disease, prevent BMESH progress to ONFH.

**Several studies have reported that CD/MDCD is effective for the management of BMESH in relieving pain and shortening natural course of BMESH** [4, 19, 22]. **Bashaireh et al.** [19] **evaluated clinical results of CD in 11 patient (11 hips) with BMESH. Pain decreased 1week after surgery in all patients. Paraskevopoulos et al.** [22] **reported that at follow-up of less than 4 weeks 16/29 (55%) had free of pain in patients underwent CD, within 1–3 months of follow-up, 47/48 (98%) had pain resolution.** Gao et al. [4] reported clinical outcomes of 36 hips using the MDCD procedure, mean time of symptom disappearance was 3.8 ± 2.9 months in patients with unilateral hip lesion. Mean symptom disappearance time of left side was 4.0 ± 2.7 months in patients with bilateral hips while right side was 4.2 ± 2.1months. A fast pain relief at rest was observed in this study. HHS was on average 93.1 ± 3.6 and 94.0 ± 3.0 at postoperative 3 and 6 months, respectively. **Postoperative pain relief time is shorter compared with those studies** [4, 19, 22]. The results of this study in HHS, VAS of BMESH were better than those of the Gao’s study [4].

Furthermore, in this study beneficial results were also obtained on MRI. Bone marrow edema completely disappeared in all hips at postoperative 3 months. **Paraskevopoulos et al.** [22]**’s results showed at 1–3 months of follow-up 40/46 (87%) in patients underwent CD had complete resolution of BMESH, from 3 to 6 months of follow-up 64/68 (94%) completely disappeared.** Gao et al. [4] reported that MRI revealed complete disappearance of bone marrow edema within 6 months after surgery. In this study, it showed more favorable results in the disappearance time of bone marrow edema compared with other studies. These outcomes may be related to the treatment of intraarticular concomitant diseases, such as hip effusion/synovitis, through arthroscopic procedure. **We reviewed the preoperative MRI T2-weighted images in Gao and Bashaireh’s studies** [4, 19] to find that there was hip effusion in the involved hips. This may account for the slower disappearance of pain and functional recovery. Mayes et al. [23] reported that the hip pain and sports/recreation function scores (Hip and Groin Outcome Scores, HAGOSs) were severer and lower in female dancers with effusion-synovitis compared to those without effusion-synovitis, respectively. Even when other factors such as BMI, age, and hypermobility were accounted for, indicating a strong relationship between hip pain and the presence of effusion-synovitis on MRI. A prospective randomized controlled study of Paschos et al [24] reported that aspiration of non- or traumatic knee effusion showed better short-term outcomes compared to non-aspiration group in pain relief, ROM and swelling. It is mainly related to decrease pain relief, possibly due to the decrease in the knee joint swelling and the reduction in the intra-articular pressure [25].

Joint effusion occurs in up to 72% of cases regardless of articular collapse, possibly secondary to ONFH-associated synovitis [14]. Similarly, BMESH is accompanied by hip effusion/synovitis, which may be secondary to BMESH-associated hip effusion/synovitis. An arthroscopic washed out and synovectomy in our cases may be of clinical benefit. Since it can drain joint effusion to reduce the capsular stress from the effusion possibly improving the blood flow to the femoral head. This may relieve pain and limited ROM which caused by increased capsular tension due to joint effusion [26]. This series of patients with hip effusion/synovitis (9 hips, 100%) showed more favorable postoperative outcomes (postoperative pain relief time and disappearance time of bone marrow edema), which may be related to the decrease of joint capsule tension after effusion drainage. Hip effusion was indicated on preoperative MRI and limited ROM were found in this series of patients, which were the main reason we chose to perform arthroscopic procedures to drain hip effusion for decreasing stress of joint capsule and relieving pain, and examine and treat other concomitant intraarticular pathologies.

The other intraarticular lesions could be associated with the results after surgery in patients with BMESH. Arthroscopic-assisted MDCD allows for the treatment of concomitant intraarticular lesions in BMESH, including labral tears and loose bodies (Table 3). Serong et al. [27] performed arthroscopy in 26 hips with radiographically proved ONFH, the outcomes showed that hip arthroscopy confirmed prevalence of intraarticular concomitant lesions in > 95% of patients with ONFH. Labral abnormalities, chondral issues and cam-type deformity were the most common. Shoji et al. [16] reported that in patients with ONFH arthroscopic examination revealed labral lesions and acetabular cartilage damage in 13 patients (32%) and 22 (54%), respectively. These pathologies could be found on preoperative imaging in only ten patients (24%) and 13 (32%), respectively. In present study, concomitant intra-articular lesions that were managed included: labral debridement in 1 hip (11.1%), cartilage debridement in 1 hip (11.1%), and loose bodies removal in 1 hip (11.1%), respectively. In addition to hip effusion/synovitis, cartilage fissure, loose bodies and labral lesions were found and treated by intraoperative arthroscopic procedure, it was not found on preoperative MRI images. Hence, preoperative images did not show the full extent of concomitant intraarticular lesions. Although there are currently no reports in the literatures, intraarticular concomitant diseases in patients with BMESH may also be present. This study showed that hip arthroscopy had an important role for finding and treating intra-articular concomitant lesions in patients with BMESH. This may be another reason for the good curative effect in this series of cases. These conditions showed that hip effusion/synovitis could affect the clinical outcomes after MDCD for BMESH. Arthroscopic procedure of hip effusion/synovitis can shorten postoperative pain relief time, disappearance time of bone marrow edema. Arthroscopic procedure can also detect and treat other concomitant intraarticular pathologies.

Additionally, we also evaluated the ability of daily living (HOS-ADL), quality of life (iHOT-12) and ROM of the hip joint in this series of patients, significant improvement was achieved postoperatively. These early-stage results (Table 2) indicate that good hip function and ROM were obtained with lower complications. In this study, wire hole hemorrhage occurred in 1 hip, bleeding stopped through cryotherapy and compression therapy after 2 dressing changes. No hip arthroscopy-related complications occurred, such as groin numbness, pudendal neurapraxia, sciatic paralysis, iatrogenic cartilage scuffing and penetration of labrum during portal establishment. No severe MDCD-related complications occurred. Comparatively, traditional standard CD using an 8-mm hollow trephine [17] has a lower complication rate, including perforation of the femoral head cartilage, subtrochanteric fracture, and weakening the subchondral bone support contributing to collapse. Arthroscopic-assisted MDCD can be a safe operation with fewer complications.

However, BMESH with hip effusion/synovitis may be associated with hip pain and progression of ONFH or osteoarthritis (OA) [5, 28], and can be treated through arthroscopic-assisted MDCD with good results. Here, we have several therapeutic suggestions for BMESH with hip effusion/synovitis. First, the reasonable surgical indications avoid overtreatment, including failure of conservative treatment, severely pain-limited ROM of hip joint, unwilling conservative treatment with requiring surgical treatment to shorten the course of the disease, severely affecting sleep during night due to pain, and clearly diagnosing hip effusion/synovitis by preoperative MRI. Second, regarding to selection of surgical method, the arthroscopic-assisted MDCD is suggested through outside-in fashion without distraction for preventing arthroscopy- and decompression-related complications [18]. Third, for the rehabilitation program, the patients are required partial weight bearing using crutches for 6 weeks postoperatively, and the operative hip avoids twisting for 3 months for preventing fracture of involved hip.

There are several limitations in this study. Firstly, this cohort analysis has a small sample size, shorter follow-up. Secondly, there was no matched group because all individuals that presented with BMESH were managed similarly and there was no cohort of single MDCD or non-surgical treatment. Finally, this was a retrospective study. To further evaluate the safety and effectiveness, it is necessary to carry out large-scale, prospective clinical studies in the future. For example, we will perform a prospective, randomized, controlled trial of MDCD with or without assisted-hip arthroscopy, arthroscopy-assisted versus aspiration-assisted MDCD to find the optimal surgical procedure with the best efficacy and least trauma. However, we believe that this study contributes to the literature by providing the first report of early-stage follow-up in patients with BMESH treated with arthroscopic-assisted MDCD.

## Conclusions

In conclusion, hip effusion/synovitis could affect the clinical outcomes after MDCD in patients with BMESH. Arthroscopic procedure of hip effusion/synovitis can shorten postoperative pain relief time, disappearance time of bone marrow edema on MRI. It can find and manage other concomitant intraarticular pathologies, and be a safe operation with fewer complications.

## Data Availability

The datasets generated and/or analysed during the current study are not publicly because we will enlarge the sample size and extend the follow-up time to further explore the relationship between hip effusion/synovitis and clinical outcome, but these are available from the corresponding author on reasonable request.

## Abbreviations

BMESH: Bone marrow edema syndrome of hip.
MDCD: Multiple drilling core decompression
